# Targeted Therapy for Pulmonary Hypertension in Premature Infants

**DOI:** 10.3390/children7080097

**Published:** 2020-08-15

**Authors:** Shannon N. Nees, Erika B. Rosenzweig, Jennifer L. Cohen, Gerson A. Valencia Villeda, Usha S. Krishnan

**Affiliations:** 1 Division of Pediatric Cardiology, Columbia University Irving Medical Center, New York, NY 10032, USA; Shannon.nees@nemours.org (S.N.N.); esb14@cumc.columbia.edu (E.B.R.); 2 Division of Pediatric Cardiology, Mount Sinai Medical Center, New York, NY 10029, USA; jennifer.cohen3@mssm.edu; 3 Division of Pediatric Cardiology, Arnold Palmer Hospital for Children, Orlando, FL 32806, USA; gerson.valenciavilleda@orlandohealth.com

**Keywords:** pulmonary hypertension, prematurity, bronchopulmonary dysplasia

## Abstract

Pulmonary hypertension (PH) is common in premature infants with bronchopulmonary dysplasia (BPD) and is associated with significant mortality. Despite expert consensus suggesting the use of targeted therapies such as phosphodiesterase inhibitors, endothelin receptor antagonists, and prostanoids, there is little data on safety and outcomes in infants with BPD-associated PH (BPD-PH) treated with these medications. We sought to describe the pharmacologic management of BPD-PH and to report outcomes at our institution. Premature infants with BPD-PH born between 2005 and 2016 were included. Follow-up data were obtained through January 2020. A total of 101 patients (61 male, 40 female) were included. Of these, 99 (98.0%) patients were treated with sildenafil, 13 (12.9%) with bosentan, 35 (34.7%) with inhaled iloprost, 12 (11.9%) with intravenous epoprostenol, and nine (8.9%) with subcutaneous treprostinil. A total of 33 (32.7%) patients died during the study period and 10 (9.9%) were secondary to severe to pulmonary hypertension. Of the surviving patients, 57 (83.8%) had follow-up data at a median of 5.1 (range 0.38–12.65) years and 44 (77.2%) were weaned off PH medications at a median 2.0 (range 0–8) years. Mortality for BPD-PH remains high mostly due to co-morbid conditions. However, for those patients that survive to discharge, PH therapies can frequently be discontinued in the first few years of life.

## 1. Introduction

Infants born prematurely are at increased risk of developing bronchopulmonary dysplasia (BPD) as well as pulmonary hypertension (PH) due to immature lung development and subsequent lung injury [1,2]. The definition of BPD has evolved and is not universally agreed upon but generally includes those infants with persistent respiratory symptoms and the need for supplemental oxygen for at least 28 days after birth and with continued need for supplemental oxygen at 36 weeks of corrected age [3,4]. PH is characterized by an increase in pulmonary vascular resistance that eventually leads to right ventricular failure. PH occurs in 14–28% of premature infants with BPD and is associated with a significant mortality of 14–47% [5,6,7,8,9,10,11,12,13]. Several drugs approved for adults with pulmonary arterial hypertension (PAH) are used off-label in these patients, including targeted therapies such as phosphodiesterase (PDE) type 5 inhibitors, endothelin receptor antagonists, and prostanoids. Recent recommendations suggest the initiation of PH-specific medication in infants with sustained PH after optimization of underlying respiratory and cardiac disease [14,15]. Although these medications are widely used in this population, there is little data on safety, tolerability, and outcomes for infants treated with these medications for PH. We sought to describe the pharmacologic management of BPD-associated PH (BPD-PH) at a single institution, Columbia University Irving Medical Center (CUIMC), and to report on clinical outcomes for these patients in the current medical era.

## 2. Materials and Methods

We performed a retrospective study to investigate the safety and tolerability of targeted pharmacologic therapies in premature infants with BPD-PH. Premature children (born < 32 weeks gestational age) with a diagnosis of PH and BPD who were treated with targeted therapy at CUIMC between 2005 and 2016 were included and follow-up data were collected through January 2020. Billing and pharmacy records were used to identify the subjects. Both infants born at CUIMC and those transferred to CUIMC for treatment were included as long as the diagnosis of PH was made prior to two years of age. Children were treated in both the neonatal intensive care unit (NICU) and pediatric intensive care unit at CUIMC. BPD was defined as the need for respiratory support with supplemental oxygen and/or positive pressure at 36 weeks corrected age. PH diagnosis was based on echocardiogram and cardiac catheterization when available and was, otherwise, based on a diagnosis by the cardiology team as some patients were diagnosed with PH and started on treatment prior to transfer to our institution. 

We reviewed reports from the echocardiogram obtained closest to the time of diagnosis and the most recent echocardiogram and collected data on PH severity, which was graded as none, mild, moderate, or severe. This was determined using estimated right ventricular (RV) pressure based on the jet of tricuspid regurgitation (TR) if available with mild being considered <50% systemic, moderate 50–75% systemic, and severe >75% systemic. If no TR, the position of the interventricular septum was used with septal flattening consistent with mild PH and transient posterior bowing of the septum consistent with moderate PH and severe posterior bowing consistent with severe PH. The presence of right ventricular hypertrophy (RVH) and direction of shunting at the patent ductus arteriosus (PDA) if present were also considered. Data from cardiac catheterization at the time of diagnosis and at the time of most recent follow-up were collected if available. There was no standard protocol for obtaining echocardiographic or catheterization data before and after starting medications and the timing of these studies was based on the decisions of the treating team.

Children who did not receive any PH treatment were not included in the study because the subjects were identified through pharmacy records. In addition, children with a diagnosis of Trisomy 21 or other genetic syndromes associated with PH, those with congenital diaphragmatic hernia and those with congenital heart disease other than an insignificant atrial or ventricular shunt or a PDA were excluded.

Data were obtained through a retrospective chart review that included both inpatient and outpatient records. Data collected included demographic information and maternal risk factors from delivery notes when available, and information on conditions associated with prematurity from progress notes and discharge summary. All decisions regarding respiratory support were made by the treating neonatology team and respiratory support was adjusted frequently in this population. Data were collected on the method of respiratory support at the time of PH diagnosis, at 36 weeks corrected gestational age and at the time of discharge from our hospital or transfer to an outside institution or rehabilitation facility. Additional details about respiratory support were not able to be obtained due to the retrospective nature of this project. The presence of necrotizing enterocolitis (NEC), either treated medically or surgically, and retinopathy of prematurity (ROP) was obtained from the chart. Intracranial pathology including intraventricular hemorrhage (IVH), periventricular leukomalacia (PVL), or any other finding associated with prematurity was obtained from review of radiology results. Sepsis was defined as the need for a course of antibiotics beyond 48 h with or without a positive culture. 

Pharmacy data were reviewed to determine which medications were initiated as well as the starting and ending date and dosing information. Treatment was based on the decisions of the clinical team and the available knowledge and medications at the time. If a medication was stopped during the stay, records were reviewed to determine the reason for stopping and whether there were any adverse side effects. The outpatient electronic medical record was reviewed to determine the most recent follow-up, current medications, and the date of stopping medications.

Statistical analyses were performed using Stata software, version 15.1 (StataCorp, College Station, Texas, USA). Clinical and demographic variables were described using standard summary statistics including a median with interquartile range and range for non-parametric data. Standard univariable analyses were used to compare patients who survived to those who died. A multivariable logistic regression was performed to determine factors associated with mortality. A *p*-value of <0.05 was considered significant. This study was approved by the Columbia University Medical Center’s Institutional Review Board with a waiver of informed consent due to the retrospective nature of the study, which involved no more than minimal risk to the subjects (IRB AAAM2970).

## 3. Results

### 3.1. Patient Population

#### 3.1.1. Birth and Pregnancy

A total of 101 patients (61 (60%) male and 40 (40%) female) were identified with BPD and PH treated at our institution. The median gestational age at birth was 25 weeks (IQR 24–27; range 23–32). Additional birth and pregnancy characteristics are described in Table 1. Twenty-four (23.8%) patients were born at Columbia and the remaining patients were transferred from an outside hospital NICU or transferred after being readmitted from home due to new or worsening symptoms. Of the 77 patients transferred or admitted from home, 59 (76.6%) had a known diagnosis of pulmonary hypertension at the time of admission.

#### 3.1.2. Pulmonary Hypertension

Pulmonary hypertension was diagnosed at a median age of 120.5 (IQR 78–176, range 2–628) days. There were 8 (7.9%) patients who were diagnosed at less than 28 days of life. A total of 87 (86.1%) patients were diagnosed with pulmonary hypertension prior to discharge from the NICU and the remaining 14 (13.9%) were diagnosed when they were readmitted to the hospital with worsening symptoms, often after a viral illness.

Echocardiographic data was recorded from the first available echocardiogram after PH diagnosis. For patients who were diagnosed at an outside hospital and then transferred, this was the first echocardiogram available at our institution. The echocardiogram was performed a median of 1 days after suspected PH diagnosis (IQR 0–19, range 0–286). The findings on that echocardiogram were consistent with severe PH in 52 (51.5%) patients, moderate PH in 20 (19.8%) patients, and mild PH in 5 (5.0%) patients. In the remaining 24 (23.8%) patients, a grade could not be determined based on the available data.

Cardiac catheterization was performed in 44 (43.6%) patients a median of 137 (IQR31–388; range 0–1352) days after PH diagnosis. Of those, 18 (17.8%) patients had more than one cardiac catheterization performed. There was no standard protocol for obtaining echocardiograms or cardiac catheterizations while initiating pulmonary hypertension medications and, since many of these patients were on PH medication prior to transfer, additional quantitative data was not included in this study.

#### 3.1.3. Associated Lesions and NICU Course

At total of 43 (42.6%) patients had an atrial communication, either an atrial septal defect or patent foramen ovale, three of whom underwent closure of their atrial septal defect. There were 2 (2.0%) patients with a small, insignificant muscular ventricular septal defect (VSD) in addition to an atrial communication. There were 75 (74.3%) patients who had a PDA and 48 (64%) of those underwent surgical ligation or transcatheter closure. Fourteen (13.9%) patients had pulmonary vein stenosis documented on an echocardiogram or cardiac catheterization. One of those patients had drug eluting stents placed and is doing well at three years of age. Another patient required surgical repair, which was complicated by restenosis and, subsequently, died of sepsis and respiratory failure. Of the remaining 12 patients, four died, seven had spontaneous resolution of mild single pulmonary vein stenosis between seven months and nine years of follow-up, and one has persistent stenosis of a single vein that is being monitored. Additional comorbidities associated with prematurity are described in Table 2 for those patients in whom detailed NICU course was available. 

Almost all patients (*n* = 98 (97.0%)) required mechanical respiratory support during their initial NICU stay and 38 (37.6%) required high frequency oscillatory ventilation. All decisions about respiratory management and adequacy of support were made by treating the NICU team and specific settings were frequently adjusted in this population. Additional details about respiratory support are provided in Table 3. A total of 33 (32.7%) patients required a tracheostomy for prolonged respiratory support.

### 3.2. Pharmacologic Treatment with Targeted PH Medications

There was no standard treatment protocol applied to all patients over the study period, but many patients received similar treatments depending on the severity of disease and their response. Table 4 describes the PH-targeted pharmacologic therapy administered to the infants during their hospitalization. Of the 101 patients, 61 (60.4%) required inhaled nitric oxide for management of PH. Not including nitric oxide, 58 (57.4%) patients were treated with only one medication, 22 (21.8%) were treated with two medications, 17 (16.8%) were treated with three medications, and four (4.0%) were treated with four medications.

#### 3.2.1. Sildenafil

Sildenafil was given to 99 (98.0%) patients starting at a median of 146.5 days. Sildenafil was typically started at 0.25–0.5 mg/kg/dose (range 0.2 to 1 mg/kg/dose) and titrated up to 1 mg/kg/dose orally every 8 h, as tolerated. If administered intravenously, the dose was decreased by 50%. Some patients admitted from outside hospitals received doses as high as 2 mg/kg/dose and up to every 6 h, but these doses were changed upon admission to our institution. Sildenafil was discontinued due to adverse effects in 7 (7.1%) patients. These included increased oxygen requirement or increased frequency of desaturations (*n* = 4), hypotension (*n* = 1), irritability (*n* = 1), and airway spasm (*n* = 1). 

#### 3.2.2. Bosentan 

Bosentan was used in 13 (12.9%) patients starting at a median age of 198.5 days. Most patients started with a dose of 1 mg/kg/dose given twice daily and increased to 2 mg/kg/dose twice daily as tolerated. No patients stopped bosentan secondary to side effects. Liver function tests were performed at baseline and twice weekly at the start and then monthly after a stable dose was achieved and as an outpatient.

#### 3.2.3. Inhaled Iloprost 

Inhaled iloprost was used in 35 (34.7%) patients starting at a median age of 188 days. The most common starting dose was 2.5 mcg/dose with a starting range from 1–7.5 mcg/dose. Dosing intervals ranged from continuous to every 4 h and were frequently titrated based on the response. Iloprost was stopped for side effects in six (17.1%) patients and these included increased oxygen requirement or increased frequency of desaturations (*n* = 4), hypotension (*n* = 1), and pulmonary edema in a patient who was subsequently diagnosed with pulmonary vein stenosis (*n* = 1). The remaining patients either improved or were transitioned to parenteral prostanoids.

#### 3.2.4. Intravenous Epoprostenol and Subcutaneous Treprostenil

Intravenous epoprostenol was used in 12 (11.9%) patients starting at a median of 234 days. The most common starting dose was 2 ng/kg/min, which was increased by 1–2 ng/kg/min every 4–6 h to 20 ng/kg/min and then much slower, as tolerated. Three patients were transitioned from intravenous epoprostenol to subcutaneous treprostenil due to access issues. Subcutaneous treprostenil was used in 9 (8.9%) patients starting at a median of 196 days of life. The most common starting dose was 1.25 ng/kg/min with increases by 2–3 ng/kg/min every 4–6 h to 20 ng/kg/min and then slower as tolerated. No patients had either epoprostenol or treprostenil discontinued due to side effects.

### 3.3. Morbidity and Mortality

A total of 33 (32.7%) patients died during the study period. Of those, 30 (90.9%) patients died before discharge from the hospital at median 189 (IQR 160–312, range 102–705) days and three (9.1%) patients died after hospital discharge at a median of 493 (IQR 406–856) days. Of the 33 deaths, 17 (51.5%) were due to respiratory failure, six (18.2%) were due to sepsis, and 10 (30.3%) were directly related to worsening pulmonary hypertension. 

On univariable analysis, none of the prenatal or birth factors including gestational age at delivery were significantly associated with mortality (Table 1). Cardiac lesions including atrial communications, VSD, pulmonary vein stenosis, or a PDA were also not significantly associated with mortality. A history of ROP (*p* = 0.028), intracranial pathology (*p* = 0.020), and sepsis (*p* = 0.043) while in the NICU were all associated with increased frequency of death on univariable analysis (Table 2).

Regarding medication use, sildenafil, treprostinil, and bosentan use was not associated with a higher frequency of mortality. Those patients who received iloprost (*p* = 0.003) or epoprostenol (*p* = 0.017) had a higher frequency of death compared to those who did not receive those medications, which may have reflected the severity of their disease and their usage at the time of escalating illness.

We performed a multivariable logistic regression for mortality, which included variables with a *p* value < 0.05: ROP, intracranial pathology, sepsis, iloprost, and epoprostenol. In addition, we included gestational age, treprostenil, and bosentan in the model. Sildenafil was not included since it was used in the majority of patients. Based on our model, only iloprost use was significantly associated with death (OR 3.59, *p* = 0.036).

### 3.4. Discharge and Follow-Up

**Figure 1:**
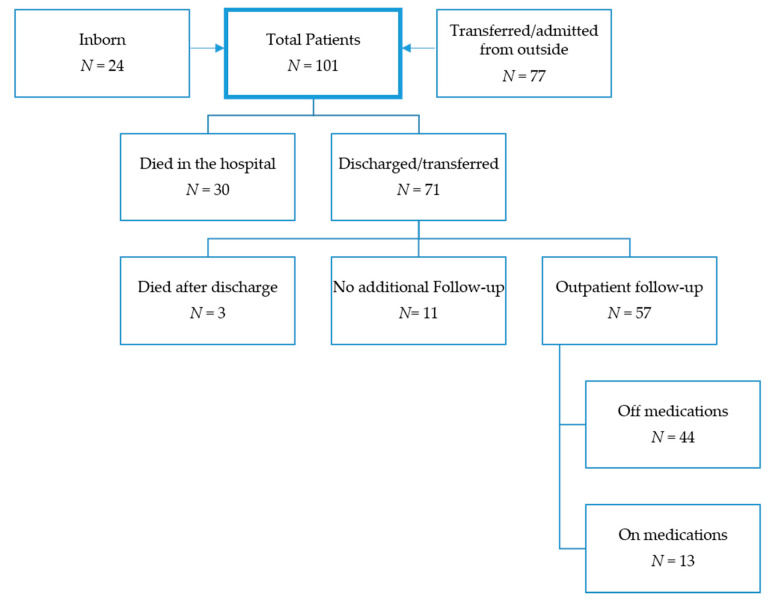
Patient flowchart.

Of the 101 patients, 71 (70.3%) were discharged or transferred from our hospital alive either from their initial NICU stay or from the hospitalization in which PH was diagnosed at a median age of 235 (IQR 168–296, range 57–663) days. A total of 22 (31.0%) patients were transferred back to an outside institution, 33 (46.5%) were discharged to a rehabilitation facility, and 16 (22.5%) were discharged home (Figure 1).

At the time of hospital discharge, 64 (90.1%) patients remained on PH medications, 49 (69.0%) patients were on one medication, 12 (16.9%) patients were on two medications, and three (4.2%) patients were on three medications. A total of 65 (91.5%) patients required respiratory support at the time of discharge or transfer (Table 3). 

Of the 68 surviving patients, 57 (83.8%) had follow-up data available after hospital discharge at a median of 5.1 (IQR 3.70–8.00, range 0.38–12.65) years. Of those 57 patients, 44 (77.2%) were weaned off PH-specific medications at a median of 2.0 (range 0–8) years. At the time of the most recent echocardiogram, 44 (77.2%) patients had no evidence of elevated right sided pressures, six (10.5%) patients had mild PH, one (1.8%) patient had moderate PH, and six (10.5%) patients had severe PH.

## 4. Discussion

Premature infants and those with low birth weight are at risk of developing BPD due to lung immaturity at the time of birth and subsequent lung injury [1,2]. In addition to the effects on the developing lungs, premature birth and subsequent changes related to mechanical ventilation also affect the normal development of the pulmonary vasculature, which leads to narrowing of the vessels, decreased vascular compliance, and vascular remodeling. This process can lead to increased pulmonary vascular resistance (PVR) and pulmonary hypertension [1,16].

The incidence of PH in infants with BPD is reported to be between 14–28% in studies with variable inclusion criteria, and there is increased frequency in infants born with lower birth weights and more severe BPD [5,6,7,8,9,10,11]. A recent multicenter retrospective study demonstrated that, when compared to premature infants without PH, those with PH had increased mortality (21% vs. 5%), and increased morbidity including prolonged ventilation, increased rates of tracheostomy and gastrostomy tube, and more frequent readmissions in the first year of life [7]. Other studies have similarly demonstrated increased mortality in children with BPD-PH compared to BPD alone with mortality rates ranging from 14–47% [8,11,17,18,19,20,21]. There is significant variation in the inclusion criteria among these studies, which likely accounts for the large variation in mortality that has been reported.

In our study, 33% of patients died during the study period. However, only 10% were directly attributable to worsening pulmonary hypertension occurring due to intercurrent illness or aspiration events. There are likely several factors contributing to the high mortality in this cohort. Our institution is a tertiary referral center, which accepts transfers from other regional centers for management of respiratory failure and PH. The majority of patients (76%) included in this study were born at other institutions and then transferred for further care with escalating illness and significant lung disease. Therefore, this cohort of patients represents the more severe spectrum of BPD-PH. The median gestational age of 25 weeks and median birth weight of 646 g indicates that these patients were at high risk of complications of prematurity. In addition, our study period ranged from 2005–2016 and there were changes in treatment of both chronic lung disease and PH over that time. Patients who were diagnosed with BPD-PH but did not receive medical treatment were not included in this study and those patients are likely to have more mild disease and lower mortality. In our study, the majority of deaths occurred during the initial NICU admission within the first year of life. For those patients who survived to discharge, the long-term survival was highly satisfactory. 

In our study, pulmonary hypertension was typically diagnosed around four months, which is a time point when infants would be expected to be close to term and improving. Therefore, it is recommended that infants who are not following the typical course be screened for PH with an echocardiogram, even if they have had a prior normal echocardiogram. In addition, pulmonary vein stenosis was found in 14% of patients in this study and has been demonstrated in an increasing number of patients with BPD-PH and is associated with increased mortality [22,23,24]. Recent guidelines suggest that all infants diagnosed with BPD should receive a screening echocardiogram at 36 weeks adjusted age with repeat echocardiograms monthly if there is continued need for respiratory support, or more frequently, if respiratory support is escalated. If PH is diagnosed or there is concern for pulmonary vein stenosis, echocardiograms should be repeated every 1–2 weeks until it is stable [14]. 

The treatment for patients with BPD-PH is not clearly defined and there is limited data to show that interventions improve long-term outcomes. The clinical course of patients with BPD-PH differs from patients with idiopathic pulmonary hypertension and other forms of secondary PAH in that many patients with BPD-PH show improvement over time [5,21]. Although lower birth weight and more severe PH are predictors of decreased survival, it is difficult to predict which patients with BPD and PH will improve. Intercurrent illnesses and aspiration pneumonias complicate parenchymal lung disease and cause worsening PH and are often the cause of significant morbidity and mortality in this population. Goals of PH treatment include increasing survival as well as minimizing other complications of prematurity, which, thereby, supports pulmonary growth and development [21]. Routine treatment involves proper nutrition, respiratory management with target saturations between 92–95%, pH balance, avoidance of respiratory infections, and treatment of contributing comorbidities including aspiration, gastroesophageal reflux disease (GERD), structural airway disease, pulmonary artery and vein stenosis, left ventricular diastolic dysfunction, and closure of cardiac shunts if they contribute to excessive pulmonary blood flow [2,14,15,16]. Inhaled nitric oxide (iNO) has been shown to acutely lower PAP in these patients and is often used as a component of the treatment regimen, especially in the acute setting [21,25]. 

Sildenafil, which is a selective PDE-5 inhibitor, is a vasodilator that can be administered orally and is approved for use in adults with PAH and commonly used off-label in children due to the potential morbidity and mortality associated with untreated PH. In a large randomized, double-blind, placebo-controlled study of oral sildenafil in pediatric patients with idiopathic PH and congenital heart disease-associated PH, initial results showed hemodynamic and functional improvement in children on medium-dose and high-dose sildenafil. However, follow-up data demonstrated an increased mortality in children receiving high dose sildenafil, which led the American Food and Drug Administration (FDA) to release a black box warning against the use of sildenafil in pediatric patients [26,27,28,29]. The Pediatric Pulmonary Hypertension Network put forth a consensus statement explaining the limitations of the follow-up extension study and the FDA warning was revised to add that healthcare providers should consider the risk and benefits of sildenafil for the individual patient [30]. This randomized trial did not include infants with BPD-PH and data in this population are even more limited. 

In a recent multicenter retrospective study, 17% of patients with BPD-PH were treated with sildenafil [31]. A retrospective review of 25 infants with chronic lung disease and PH showed hemodynamic improvement in 88% of patients receiving long-term sildenafil and the medication was well-tolerated [17]. Several other small retrospective studies have demonstrated improvement in echocardiographic measurements of PH after sildenafil initiation [19,32,33,34,35]. A recent meta-analysis studied the effectiveness and safety of chronic use sildenafil in preterm infants with BPD-PH and found an overall mortality of 29.7% per year and demonstrated improvement in estimated pulmonary arterial pressure and respiratory severity scores in patients on sildenafil [36]. Our group previously reported on our experience with sildenafil in children with PH including those with BPD-PH and demonstrated that PH improved over time in these patients and sildenafil was well-tolerated [37].

The majority of patients (98%) in this study received sildenafil and 62% remained on it at the time of discharge from the hospital. Overall, it was well-tolerated with 7% of patients needing to discontinue the medication due to adverse effects in which none were life-threatening. Other studies have reported transient hypotension as a frequent side effect of sildenafil in this population as well as increased incidence of GERD [19,38]. Given that it is easy to administer and relatively well tolerated, at this time, sildenafil is a good initial choice for therapy in infants with BPD-PH.

Bosentan is a nonselective endothelin receptor antagonist and is currently the only FDA-approved targeted PH medication in children, but it is approved only for children older than 3 years. A small case series demonstrated hemodynamic and symptomatic improvement over the course of several years in six patients with BPD-PH, including four of whom were also treated with sildenafil [39]. It was used infrequently in our patients and generally as an add-on therapy because of the need for liver function monitoring. The survival was higher in patients treated with bosentan compared to other medications, but this is likely related to patient selection. Given the lack of significant side effects and association with improved outcomes, Bosentan is a reasonable choice for add-on therapy in this population.

Iloprost is a prostacyclin-analog approved for use in adults that has been shown to be effective in decreasing mean airway pressure and improving functional class in children with PAH [40]. Inhaled iloprost has been shown to improve oxygenation and pulmonary artery pressure in a small series of premature neonates with impending respiratory failure, but larger studies in this population are lacking [41]. The use of inhaled iloprost in our study was significantly associated with increased mortality in a multivariable model. This is likely reflective of the severity of disease in the cohort of patients in whom iloprost was initiated. Iloprost is known to be associated with a risk of bronchospasm and had the highest frequency of side-effects in our study with 17% of patients discontinuing use, which is most commonly due to increased oxygen requirement [40]. Its rapid onset of action and ease of discontinuation make it a convenient choice for patients who are having frequent desaturations and pulmonary hypertensive crises and it can be considered in selected infants with severe PH as a bridge to transition to parenteral prostacyclins.

There are very limited data on the use of continuous prostacyclins in infants with BPD-PH. A case report in 2005 demonstrated improvement in pulmonary artery pressures, quality of life, and eventual discontinuation of home ventilation in a child with BPD-PH treated with IV epopostenol and another case report demonstrated improvement in RV systolic pressures [42,43]. Although prostanoids are not FDA approved in this population, they were used in our cohort for patients in whom other therapies were not sufficient. Nine patients were discharged or transferred from our hospital on prostacyclins and they were very well-tolerated with no patients discontinuing them due to side-effects. Intravenous access can prove to be a significant challenge in premature infants, especially those who have had multiple procedures and central lines. Therefore, subcutaneous treprostinil, which was used in 9% of our patients, can be a good option to avoid further vascular injury and safely deliver the medications. Our group previously reported our results with the first five infants with chronic lung diseasesuccessfully treated with subcutaneous treprostinil with improvement in the echocardiographic assessment of PH and decreased need for respiratory support after initiation of therapy [44]. 

Ideally, treatment of these patients would benefit from larger randomized clinical trials. However, barriers to clinical trials include difficulty identifying clinically meaningful outcomes in pediatric patients, recruiting sufficient numbers of patients, the need for long-term follow-up to assess neurodevelopmental outcomes and side effects and ethical concerns surrounding infants and children in clinical trials, particularly when evaluation involves tests such as cardiac catheterization and echocardiography which may require sedation [45]. Multi-center prospective trials studying the use of these medications in premature infants will be critical to determine medication efficacy in the future. Given that premature infants who develop pulmonary hypertension tend to have other significant comorbidities, this is a high-risk group that will benefit from medication optimization. For those patients who can be supported through the most critical period, there is a high likelihood of improvement or resolution of pulmonary hypertension over time, as demonstrated by the fact that 88% of patients with follow-up in our study had either mild or no evidence of PH on their most recent echocardiogram and 77% were weaned off of all PH medications.

There are several important limitations of this study. As a retrospective review, the data available were limited to what was recorded in the electronic medical record. Both assessment and treatment were based on the decisions of the clinical team and the available knowledge and medications at the time. Although optimization of respiratory support is critical for management of BPD-PH, detailed data on respiratory support settings and adequacy of support were not able to be collected. There was no control group who did not receive medical therapy and, therefore, it is difficult to determine how the medications affected outcomes. Since pharmacy records were used to identify subjects, we may have missed those who were observed with transient or mild PH and did not receive treatment. In order to provide medium to long-term follow-up data, we only included children born through 2016. As treatment for chronic lung disease continues to evolve, outcomes for children with BPD-PH will hopefully continue to improve.

## 5. Conclusions

Targeted PH therapy was well tolerated among infants with BPD-PH. We believe that pharmacologic treatment of BPH-PH in infants is safe and effective and should be used alongside optimal treatment of underlying lung disease and other comorbidities (aspiration, GERD, structural airway disease, pulmonary artery stenosis, pulmonary vein stenosis, left ventricular diastolic dysfunction, and cardiac shunts). Targeted medications should be reserved for those patients with evidence of elevated pulmonary vascular resistance and right ventricular impairment consisting of at least moderate right ventricular hypertrophy or dysfunction in whom respiratory support has been optimized without improvement. The practice at our institution has evolved over the years but, typically, we utilize nitric oxide for those patients with severe PH crises who need a rapid-onset medication. For patients with mild or moderate disease, sildenafil is our typical first-line therapy with the addition of bosentan as a second line in cases where there is still continued evidence of increased right-sided pressures. The addition of a second medication often triggers a cardiac catheterization to evaluate the PH and rule out other complicating or treatable etiologies. In patients with severe disease who cannot be weaned off inhaled iloprost or who are not well controlled on oral medications alone, either intravenous epoprostenol or subcutaneous treprostenil is used based on the availability of IV access. Once medications are initiated, frequent monitoring should be performed with serial echocardiograms and brain natriuretic peptide (BNP) levels as well as cardiac catheterization as needed. For patients who are discharged home on medications, outpatient follow-up is recommended every 3–4 months with regular echocardiographic and biomarker assessment and medications are weaned off when symptoms and right sided pressures are improved. Mortality for BPD-PH in this cohort is still high, often from co-morbidities. However, for those patients that survive to discharge, their subsequent survival is good and PH therapies can often be weaned off in the first few years of life.

## Figures and Tables

**Table 1:** Pregnancy and birth characteristics.

	All Patients *n* = 101	Survived *n* = 68	Died *n* = 33	*p*
Sex				0.976
Male	61 (60.4%)	41 (60.3%)	20 (60.6%)	
Female	40 (39.6%)	27 (39.7%)	13 (39.4%)	
Multiple Gestation	25 (24.8%)	18 (26.5%)	7 (21.2%)	0.566
Type of Delivery				0.803
Vaginal	21 (20.8%)	14 (20.6%)	7 (21.2%)	
C-section	61 (60.4%)	40 (58.8%)	21 (63.6%)	
Unknown	19 (18.8%)	14 (20.6%)	5 (15.2%)	
Born outside hospital	79 (78.2%)	49 (72.1%)	28 (84.8%)	0.157
Gestational age (weeks)	25 (IQR 24–27, range 23–32)	26 (IQR 24–27, range 23–32)	25 (IQR 24–26, range 23–30)	0.428
Birth weight (grams) *n* = 78	646 (IQR 540–790, range 270–1790)	663 (IQR 550–840, range 378–1790)	587 (IQR 485–728, range 270–1335)	0.065
Prenatal steroids *n* = 54	50/54 (92.6%)	32/35 (91.4%)	18/19 (94.7%)	1.00 *
Oligohydramnios *n* = 62	6/62 (9.7%)	4/43 (9.3%)	2/19 (10.5%)	1.00 *
Chorioamnionitis *n* = 65	9/65 (13.9%)	5/43 (11.6%)	4/22 (18.2%)	0.473 *
Maternal hypertension *n* = 67	14/67 (20.9%)	10/46 (21.7%)	4/21 (19.1%)	1.00 *
Surfactant *n* = 62	50/62 (80.7%)	31/40 (77.5%)	19/22 (86.4%)	0.512 *

Numbers represent medians (interquartile range (IQR), range) for non-normally distributed continuous variables and numbers (%) for categorical variables. In cases where data were not available for all patients, the frequency and total number in that category with data available are listed. *p*-values for either chi-square or * Fisher’s exact for categorical variables and Kruskal-Wallis for non-normal continuous variables comparing those who died to those who survived.

**Table 2:** Associated findings and comorbidities.

	Total *n* = 101	Survived *n* = 68	Died *n* = 33	*p*-Value
Atrial communication	43 (42.6%)	25 (36.8%)	18 (54.5%)	0.090
Small ventricular septal defect	2 (2.00%)	1 (1.5%)	1 (3.0%)	0.549 *
Pulmonary vein stenosis	14 (13.9%)	9 (13.2%)	5 (55.2%)	0.768 *
Patent ductus arteriosus	75 (74.3%)	50 (73.5%)	25 (75.8%)	0.810
Ligated/occluded	48 (64%)	34 (50.0%)	14 (42.4%)	0.307
Retinopathy of prematurity *n* = 91	73/91 (80.2%)	45/61 (73.8%)	28/30 (93.3%)	**0.028**
Intracranial pathology *n* = 96	53/96 (55.2%)	30/64 (46.9%)	23/32 (71.9%)	**0.020**
Necrotizing enterocolitis *n* = 97	31/97 (32.0%)	19/65 (29.2%)	12/32 (37.5%)	0.412
Sepsis *n* = 93	77/93 (82.8%)	47/61 (77.1%)	30/32 (93.8%)	**0.043**

Columns represent numbers (%). For patients in whom detailed NICU course was not available, data were only included if there was specific mention of the presence or absence of a finding in the discharge summary. Total numbers included for each category are listed in the first column. In cases where data were not available for all patients, the frequency and total number in that category with data available are listed. *p*-values for chi-square or * Fisher’s exact when appropriate. The bold represents significant *p* values < 0.05.

**Table 3:** Respiratory support.

Respiratory Support at Pulmonary Hypertension Diagnosis	Number of Patients (%)
Room air	1 (1.0%)
Nasal cannula	11 (10.9%)
Continuous positive airway pressure (CPAP)	34 (33.7%)
Mechanical ventilator	38 (37.6%)
High frequency oscillatory ventilator	2 (2.0%)
Unknown	15 (14.9%)
**Respiratory Support at 36 weeks corrected**	
Nasal Cannula	3 (3.0%)
Continuous positive airway pressure (CPAP)	36 (35.6%)
Mechanical ventilator	37 (36.6%)
Unknown (but at least on supplemental oxygen) *	25 (24.8%)
Respiratory support at discharge or transfer (N = 71) **	
Room air	6 (8.5%)
Nasal Cannula	21 (29.6%)
CPAP or Bilevel positive airway pressure (BiPAP)	21 (29.6%)
Mechanical ventilator	3 (4.2%)
Tracheostomy	20 (28.2%)

* For some patients who were transferred or admitted from outside, specific details of their NICU course was not available. All patients were confirmed to need prolonged respiratory support with at least supplemental oxygen beyond 36 weeks of corrected age. ** Indicates the type of respiratory support at the time of discharge from our hospital or transfer to a rehabilitation facility or outside hospital. Twenty-two (31.0%) patients were transferred back to an outside institution, 33 (46.5%) were discharged to a rehabilitation facility, and 16 (22.5%) were discharged at home.

**Table 4:** Pharmacologic therapy.

Medication (Method of Administration)	Patients Treated N(%)	Median Starting Age in Days (IQR, Range)	Typical Dosing	Died N (% of Total pts Receiving Med)	Patients on Medication at Discharge/Transfer N(% of Total Patients Receiving Med)	Stopped Medication for Side Effect N (%)
Sildenafil (PO/IV)	99 (98.0%)	146.5 (IQR 101–175, range 39–637)	Starting: 0.25–0.5 mg/kg/dose PO q8hrs (1/2 that dose if IV) Goal: 1 mg/kg/dose PO q8hrs(0.5 mg/kg/dose IV q8hrs)	29 (29.3%)	62 (62.3%)	7 (7.1%)
Bosentan (PO)	13 (12.9%)	198.5 (IQR 172–168, range 122–406)	Starting: 1 mg/kg/dose q12hrs Goal: 2 mg/kg/dose q12hrs	1 (7.7%)	10 (76.9%)	0
Iloprost (inhaled)	35 (34.7%)	188 (IQR 148–261; range 16–426)	Starting: 1–2.5 mcg/dose q1–4 hrs (or continuous) Max: 2.5–7.5 mcg/dose	16 (45.7%)	1 (2.9%)	6 (17.1%)
Epoprostenol (continuous intravenous)	12 (11.9%)	234 (IQR 197.5–315.5, range 97–533)	Starting: 2 ng/kg/min Max: 8–46 ng/kg/min	6 (50%)	4 (33.3%)	0
Treprostinil (continuous subcutaneous)	9 (8.9%)	196 (IQR 165–216, range 135–368)	Starting: 1.25 ng/kg/min Max: 14–74.5 ng/kg/min	4 (44.4%)	5 (55.6%)	0

PO = oral, IV = intravenous, IQR = interquartile range.
